# *CTRC* gene polymorphism (p.G60=; c.180 C > T) in acute pancreatitis

**DOI:** 10.1186/s12876-016-0566-5

**Published:** 2017-01-17

**Authors:** Dorota Koziel, Stanislaw Gluszek, Artur Kowalik, Malgorzata Chlopek

**Affiliations:** 1 Faculty of Medicine and Health Sciences, Jan Kochanowski University, Kielce, Poland; 2 Clinic General Oncological and Endocrinological Surgery, Regional Hospital, Kielce, Poland; 3 Department of Molecular Diagnostics, Holy Cross Cancer Centre, Kielce, Poland

**Keywords:** Acute pancreatitis, *CTRC* polymorphism, Etiology

## Abstract

**Background:**

The aim of the study was to determine the relationship between the presence of p.G60 = polymorphism (c.180C > T; rs497078) *CTRC* and the incidence together with the clinical course of acute pancreatitis (AP).

**Methods:**

Two hundred ninety-nine people suffering from AP and 417 healthy volunteers were subjected to the study. DNA was isolated from blood samples.

**Results:**

*CTRC* p.G60 = polymorphism (c.180C > T) occurred more frequently in the AP group (*p* = 0.015). The CT and TT genotype was found in 27.8% of the AP patients and in 19.9% of the healthy subjects (*p* = 0.017). No significant correlation was found between having the CT and TT genotype and the severity of the AP clinical course. In 6 patients (2%) with the CT genotype, a *SPINK1* gene mutation was found, while in the control group it was found in 3 patients (0.7%), (*p* > 0.05). All patients with the present *SPINK1* mutation with the CT genotype had a moderate or a severe course of the disease (*p* = 0.0007).

**Conclusions:**

*CTRC* polymorphism Hetero p.G60=; c.180C > T increases the risk of an AP occurrence and together with the *SPINK 1* mutation, may be responsible for a more severe course of the disease.

## Background

In recent years, a higher incidence of acute pancreatitis (AP) has been reported in many countries. Poland is one of the countries with a high incidence rate, which equals 79,7/100000 [1].

It is generally accepted that the direct cause of the disease is the activation of digestive enzymes in the acinar cells. An inflammatory condition in the pancreatic area initiates a cascade of immunological events, which play a significant role in disease development [2]. The course of the disease is usually mild, but in about 20% of patients, moderate or severe AP develops [1]. The most common causes of AP include cholelithiasis and alcohol consumption, however some studies point to the importance of genetic mutations and polymorphisms [3–6]. Such an approach indicates a complex and multifactorial nature of acute pancreatitis. Genetic factors should be taken into account together with other factors which cause the disease [7]. So far, there is no long term follow-up that would allow us to understand the role of genetic factors during the first onset of AP [8].

Some mutations play an important role in the development of pancreatitis. In normal conditions (genes are not mutated), trypsin in the pancreatic parenchyma is not activated, whereas mutations of genes cause disorders in secretion, enzymes are activated and the mutations predispose the pancreas to pancreatitis. These factors include: the gene coding cationic trypsinogen (*PRSSI*) and serine peptidase inhibitor Kazal type 1 (*SPINK1*). Other genes regulating trypsin, such as the independent calcium-sensing receptor gene (*CASR*), the gammaglutamyltransferase1 gene (*GGT1*), *the* cystic fibrosis transmembrane conductance regulator (*CFTR*) and the recently discovered chymotrypsin C gene (*CTRC*), seem to be the disease modifiers with a lower risk. The protein *CTRC* codes chymotrypsin C, which is produced by the pacreatic acinar cells [7].

Several papers have been published by Sahin-Toth’s group elucidating the complex regulatory function of CTRC on trypsinogen activation. Namely, trypsinogen activation in active trypsin is controlled by two processes. Firstly, the cleavage of the trypsinogen activation peptide accelerates the autoactivation of trypsinogen. The second mechanism is based on the cleavage of the peptide in the trypsinogen calcium binding loop which promotes its degradation [9–13].

Based on the GTex site (http://www.gtexportal.org/home/), data concerning the rs497078 SNP QTL, the TT genotype correlated with a lower expression of the *CTRC* gene in comparison to the CT and CC genotype in normal pancreas. However, it should be mentioned that these results are based on 149 cases with the following genotype distributions CC: 113, CT:33, TT:3. Bearing the above in mind, and taking into consideration the complex nature of, the regulation of trypsinogen activation by *CTRC*, it is possible that the T allel could be a risk factor for pancreatitis. However, in order to reveal the true function of the T allel, more studies are needed [14].

The aim of the study was to determine the relationship between the presence of the p.G60 = polymorphism (c.180C > T; rs497078, NM_007272.2:c.180C > T), *CTRC* and the incidence and clinical course of acute pancreatitis in the Polish cohort.

## Methods

The study included 299 patients with acute pancreatitis, being inhabitants of the Kielce Region in Poland who gave their informed consent for the collection of genetic material. The control group consisted of 417 healthy inhabitants who accepted the invitation to take part in the research.

The diagnosis of mild, moderate or severe acute pancreatitis (SAP), according to the Atlanta 2012 classification, was the criterion for inclusion in the study. A diagnosis of AP was based on the joint interpretation of medical history, a physical examination and targeted laboratory tests. The diagnosis was based upon the satisfaction of at least 2 of the following 3 criteria: (1) upper abdominal pain of sudden onset, frequently radiating towards the back; (2) lipase or amylase activity in serum > 3 times the upper limit of normal; and (3) results of imaging tests that allow one to obtain cross-sectional images: computed tomography (CT), nuclear magnetic resonance (NMR), or ultrasonography (USG). In the analysis of the etiology of AP, taking a person’s medical history can determine the following: alcohol consumption, the occurrence of concomitant diseases, drug intake, procedures undergone and endoscopic retrograde cholangiopancreatography (ERCP). Biliary etiology was confirmed based on USG, CT, NMR, and/or ERCP, only if there were indications for the performance of these procedures, for example, icterus. Endoscopic ultrasonography was performed in some patients to confirm biliary AP. Alcohol consumption was evaluated with the Short Alcohol Dependence Data (SADD) Questionnaire, and self-estimated alcohol consumption via an interview. Acute pancreatitis was diagnosed if a patient gained ≥10 points on the SADD Questionnaire, or if the period of alcohol abuse lasted ≥1 year, and the daily dose was 40 g of pure ethanol.

Exclusion criteria were a diagnosis (which took place during interviews) of chronic pancreatitis, as well as the enlargement of the Wirsung duct and calcifications larger than 5 mm, which were diagnosed during transabdominal ultrasonography.

The control group included adult volunteers selected by random sampling, without any apparent concomitant diseases that could have affected the structure and expression of genes to be tested in the study.

The study was approved by the Committee on Bioethics at the Faculty of Medicine and Health Sciences, Jan Kochanowski University in Kielce. Each patient and member of the control group gave their informed consent to genetic testing.

### Study samples

Whole blood samples were submitted to the Department of Molecular Diagnostics (Holycross Cancer Center, Kielce, Poland) for genetic testing.

### DNA Extraction

Whole blood extraction was performed using the Genomic Blood AX Micro Gravity (A & A Biotechnology, Gdańsk, Poland) purification system, which uses gravity technology. The DNA was extracted manually with a volume of 0.1 ml of blood, then the DNA concentration was measured using the Nano Drop (Thermo Scientific, Waltham, MA, USA).

### HRM

This PCR (forward 5'-CTGACACACAGCCCTCCC −3 'and reverse 5'-ATGGCCAGGTCTCAGGGTAT -3') for the amplification of the target sequence (162 bp) in the *CTRC* gene were designed using Primer3 web software (RefSeq NM_007272.2).

Similarly, the following primers were (F-TTGCTATGAACTCAAGAATGGAGA;

R-CCGATTTTCAAAACATAACACTG) projected for the amplification of *SPINK1*.

HRM reactions were performed using reagents from the Qiagen and the Qiagen Rotor Gene machine.

The reactions were carried out using a 15ul volume according to the following mixture: polymerase -5ul (Cat. No. 206546), water -7ul, primer F-1ul (final concentration 0,67uM), primer R -1ul (final concentration 0,67uM), DNA - 1ul (20–50 ng) /per sample.

Amplifications were performed using a 5-min initial denaturation at 95 °C followed by 37 cycles of 10 s at 95 °C, 30 s at 65 °C, with an initial 10 cycles of touchdown (1^0^/cycle) and 10 s at 72 °C.

Then the melting step was carried out in the range of 80^0^ to 93^0^ for CTRC, and 75–80 °C for exon 3 *SPINK1*. The HRM of studied samples was done in parallel with control WT. Then, all the studied samples were analyzed relative to the control curve (WT).

**Fig. 1:**
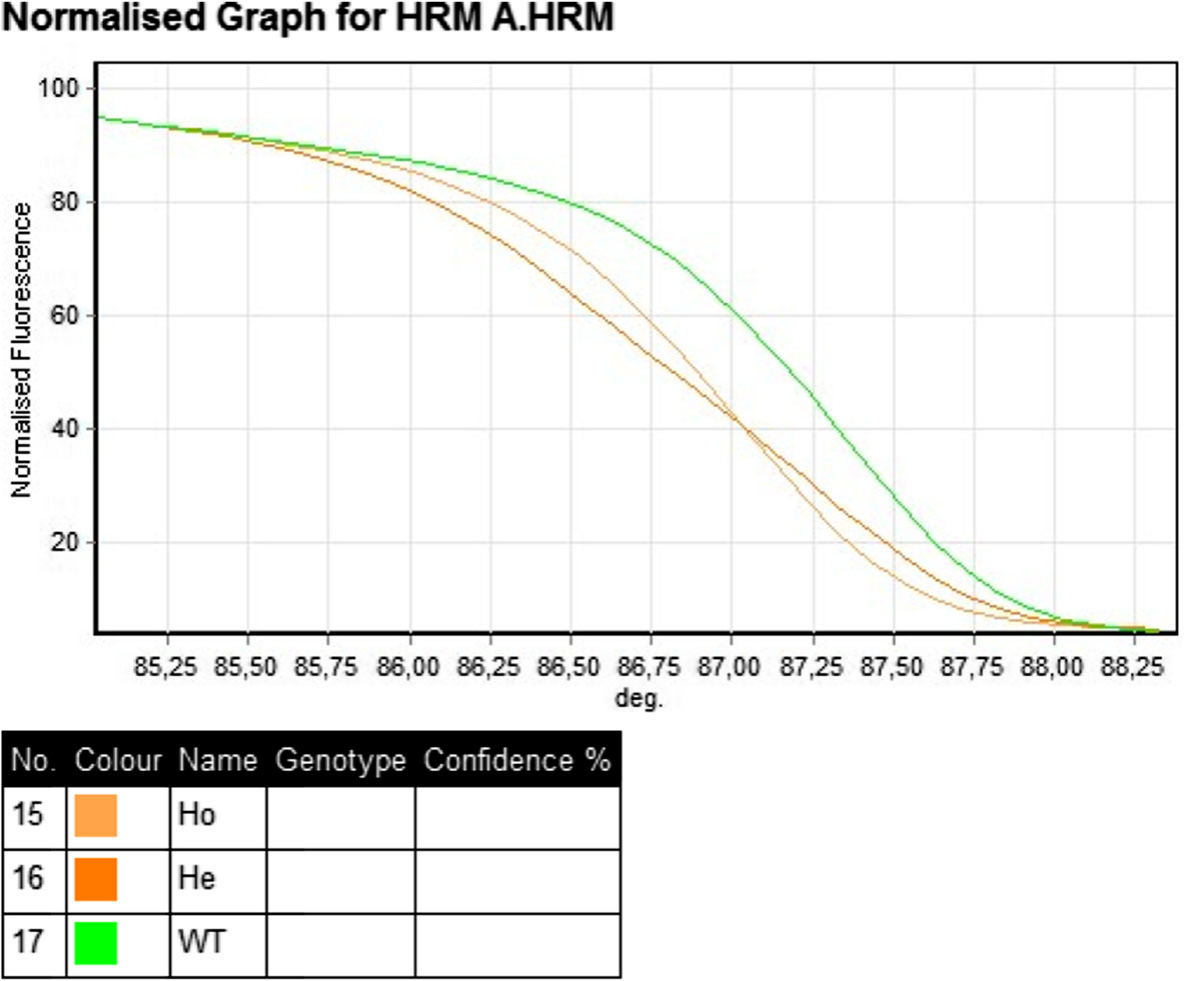
Example of HRM analysis. *Green curve* – CC genotype (c.180C), *orange* – CT genotype (c.180C > T), *light orange* – TT genotype (c.180 T)

Genotypes were assigned based on shape differences between curves. The samples with the greatest vertical distance from the WT melting curves were heterozygotes, and melting curves between the heterozygous and WT samples were homozygotes (Fig. 1).

We have comprehensively validated our HRM methodology. 20% (143 samples) of all the studied samples have been sequenced. The concordance with genotypes assigned based on melting method was 100%. Finally, our results are supported by a similar frequency of mutation detected in a healthy population in comparison to data from the 1000Genome project.

### Sequencing

The curves diverging in shape from the control curve (WT) were verified by applying capillary sequencing. The concentration of DNA in these samples was measured using the electrophoresis device Multina (Shimadzu, Japan). For the Sanger sequencing reaction, PCR amplification products were purified using 10 U (Cat. No. EN 0582) of exonuclease I and 1 U of phosphatase Fast-AP (Cat. No. EF 0651) (Thermo Fisher). The reaction was incubated for 15 min at 37 °C, followed by 20 min at 80 °C.

Sequencing reactions were performed using forward and reverse sequence-specific primers (described above) and the ABI-PRISM Big Dye Terminator version 3.1 kit (Applied Biosystems, Foster, CA; Cat. No. 4337450), according to the manufacturer’s instruction manual. Sequencing reactions were analyzed using the 3130 Capillary Sequencer (Applied Biosystems, Foster, CA). The generated sequences were compared relative to the reference sequence RefSeq (NM_007272.2), using the NCBI Blast Nucleotide program.

### Statistical methods

Categorical data were expressed as number and percentage distributions. Proportions were compared between groups by the chi-square test or the Fisher’s exact test as appropriate. Odds ratios (OR) with 95% confidence intervals (95% CI) were calculated using the univariate logistic regression model. Quantitative data were characterized by medians, and the non-parametric Mann–Whitney U test was used for comparisons between groups. The two tailed *p*-value <0.05 was considered to be statistically significant.

All computations were performed using the statistical package R, version 3.1.2 (R Core Team (2014). R: A language and environment for statistical computing. R Foundation for Statistical Computing, Vienna, Austria. URL http://www.R-project.org/.) and StatSoft, Inc. (2014). STATISTICA (data analysis software system), version 12. www.statsoft.com.

## Results

The study group included 299 patients who had acute pancreatitis (127 female, 172 male); the median age being 56 years (62 in women, 53 in men). The course of AP was mild in 69.6% of patients, moderately severe in 13.4%, and severe in 17.0%. The dominant etiological factors of the disease was cholelithiasis (41.8%) and alcohol (30.8%), but in 25.4% of the patients no other cause than an idiopathic cause of incidence was identified.

The control group consisted of 417 healthy inhabitants of the Kielce Region (271 women and 146 men), with the median age being 46 (46 in women, 48 in men, in a general good state of health).

Both in the test group, as well as in the control group, no differences in the age distribution was observed between the patients with the CT and TT, versus the CC genotype. In the test group, the median of the patients’ age with the CC genotype was 57 years, and with the CT and TT genotype, 53 years (*p* = 0.22). In the control group, the median age of the patients with the CC genotype was 47 years, and with the CT and TT genotype, 45 years (*p* = 0.68).

The CT and TT genotype was found in 27.8% of the AP patients and in 19.9% of the healthy subjects (*p* = 0.017). The frequency of the TT genotype occurrence was not statistically different between the groups of healthy and sick individuals (*p* = 0.803). However, the CT genotype was significantly more frequent in the AP group compared with the control group (*p* = 0.015) (Table 1).

**Table 1:** The comparison of the frequency of the occurrence of the *CTRC* genotype (p.G60=; c.180C, c.180 T, c.180C > T) in the AP patients’ group and the control group of healthy individuals

	Patients with AP			Control group of healthy individuals			*P* value*	*P* value**	OR	95% CI
	*N* = 299			*N* = 417						
	Female	Male	*Totals*	Female	Male	*Totals*				
*CTRC Polymorphism Homo p.G60=; c.180 T*	1 (0.8%)	4 (2.3%)	5 (1.7%)	4 (1.5%)	2 (1.4%)	6 (1.4%)	0.042	0.803	1.2	0.4-3.9
*CTRC Polymorphism Hetero p.G60=; c.180C > T*	37 (29.1%)	41 (23.8%)	78 (26.1%)	49 (18.1%)	28 (19.2%)	77 (18.5%)		0.015	1.6	1.1-2.2
*CTRC Polymorphism Homo p.G60=; c.180C*	89 (70.1%)	127 (73.8%)	216 (72.2%)	218 (80.4%)	116 (79.5%)	334 (80.1%)		0.014	0.6	0.5-0.9

*p value for the difference in genotype distribution among AP patients and control group of healthy individuals

**to compare *Totals*

*AP* acute pancreatitis, *CTRC* chymotrypsinogen C, *OR* odds ratio

To reinforce our analysis, we performed a comparison of allelic distribution between our control group and other European populations (Utah residents with Northern and Western European ancestry, Finnish, British, Iberian, Toscani) using data from the 1000 Genomes project [15]. We found that the allelic distribution in our control group is similar to the Toscani and Finish populations (T:10%, C:90%) (*p* = 0.518), but is different when we take into account the Utah, British and Iberian populations (T:8%, C:92%). The genotype distribution was in agreement with the Hardy –Weinberg equilibrium for cases (*p* = 0.50) and controls (*p* = 0.52). The T allele was the minor allele, and was observed more frequently in cases than controls (14.7% vs. 10.7%, *p* = 0.023).

In order to analyze allelic distribution in the studied population, we applied a few genetic models (Table 2.)

**Table 2:** Genetic models and allelic distribution in pancreatitis population

Genetic model	Comparison	OR	95% CI	*p*-value
Allelic	T vs. C	1.44	1.04-2.01	0.023
Genotypic	CT vs. CC	1.57	1.10-2.24	0.014
	TT vs. CC	1.29	0.39-4.27	0.68
Dominant	CT/TT vs. CC	1.55	1.09-2.19	0.014
Recessive	TT vs. CT/CC	1.17	0.35-3.85	0.80

With the four models, after applying the Bonferroni corrections to the results of the p-value, less than 0.05/4 = 0.0125 are found to be statistically significant. It should, however, be kept in mind that this amendment is very conservative, so one can assume that the results of the *p*-value = 0.014 are on the border of statistical significance. We found that after applying the Bonferroni correction, only the genotypic and dominant model are on the border of statistical significance.

The presence of the CT and TT genotype was not statistically significantly dependent on gender, neither in the test group (women: 38/127 (29.9%) vs. men: 45/172 [26.2%], *p* = 0.56, test chi^2^), nor in the control group (women: 53/271 [19.6%] vs. men: 30/146 [20.5%], *p* = 0.91, test chi^2^). The patients’ age average with the CT and TT genotype was similar, both in the test group (45.2 vs. 45.5) as well as in the control group (54.1 vs. 55.3).

Only in one case of an AP patient with the CT genotype (p.G60=; c.180C > T), was the occurrence of pancreatic disease mentioned in the family history (AP).

No significant dependency was found between having the CT and TT genotype and the severity of the AP clinical course, but in patients with the TT genotype (p.G60=; c.180 T), no severe AP was observed, which is contrary to the patients with the CT genotype (25% of whom experienced a moderate course of the disease and 27.5% a severe course of the disease; *p* > 0.05) (Table 3).

**Table 3:** The frequency of the occurrence of the *CTRC* gene polymorphisms (p.G60=; c.180C > T) in the acute pancreatitis patients depending on the severity of the clinical course, etiology and disease relapses

Analysis p.G60 = (c.180C > T; rs497078)	*No. 299*	TT No. 5	CT No. 78	CC No. 216	TT vs. CC	CT vs. CC	TT/CT vs. CC
					*P* value	*P* value	*P* value
Severity of the course							
Mild AP	208	5 (2.4%)	54 (26%)	149 (71.6%)	0.801	0.979	0.908
Moderate AP	40	-	10 (25%)	30 (75%)			
Severe AP	51	-	14 (27.5%)	37 (72.5%)			
Etiology of AP							
Alcohol	92	3 (3.3%)	21(22.8%)	68 (73.9%	0.418	0.521	0.661
Gallstones	125	2 (1.6%)	33 (26.4%)	90 (72%)			
Idiopathic	76		21 (27.6%)	55 (72.4%)			
Cancer	6		3 (50%)	3 (50%)			
Reccurrences of AP							
Yes	111	3 (2.7%)	31 (27.9%)	77 (69.4%)	0.355	0.584	0.473

*AP* acute pancreatitis, *CTRC* chymotrypsinogen C

The confirmed cause of the incidences was not related to the CT and TT genotypes, except for the cases in which a carcinoma was diagnosed in the AP course. In this group of sick subjects, 50% of the cases concerned patients with the CT genotype (p.G60=; c.180C > T). Homo (p.G60=; c.180 T) *CTRC* polymorphism was found only in 3.3% of patients with alcohol etiology (Table 3). Alcohol was also a frequent cause of AP relapses in patients with the TT genotype: 2/3 of the cases (66.7%) vs. 50.6% patients with the CC and 38.7% with the CT genotype.

Recurrent AP (RAP) was more frequent in patients with the CT and TT genotype compared with the group without relapses, differently from the CC genotype patients, but statistical significance was not confirmed (*p* = 0.47) (Table 3).

The relationship between the polymorphism of *CTRC* Hetero p.G60=; c.180 C > T (CT), known as the AP genetic risk factors, the *SPINK 1* (p.N34S) gene mutations was analyzed. In six patient (2%) with the CT genotype, a *SPINK1*gene mutation was found (p.N34S), while in the control group it was found in 3 patients (0.7%), (*p* > 0.05). All patients with the present *SPINK1* (p.N34S) mutation with the CT genotype had a moderate or a severe course of the disease (*p* = 0.0007) (Table 4).

**Table 4:** The description of the AP patients with the confirmed *SPINK 1* (p.N34S) mutation and *CTRC* polymorphism hetero p.G60=; c.180C > T (CT)

Severity of the course			Reccurrences of AP		Etiology of AP				Pancreatic diseases in family	
Mild AP	Moderate AP	Severe AP	Yes	No	Alcohol	Gallstones	Idiopathic	Cancer	Yes	No
Patient No.	*SPINK 1* mutation and *CTRC Polymorphism Hetero p.G60=; c.180C > T* (CT) *n = 6/299 (2%)*									
1	X			X		X				X
2	X			X		X				X
3	X		X		X					X
4	-	X		X			X			X
5	X		X					X		X
6		X		X		X			X	

*AP* acute pancreatitis, *CTRC* chymotrypsinogen C, *SPINK 1* pancreatic secretory trypsin inhibitor gene

A moderate or severe course of the disease was observed in 6/6 of the patients with the *SPINK1* (p.N34S) mutation and the *CTRC* CT genotype vs. 8/15 of the patients with the *SPINK1* (p.N34S) mutation and the CC genotype (*p* = 0.06 in the Fisher’s exact test). The observed correlation at the edge of statistical significance suggests that increasing the size of the group may confirm a more severe course of the disease in the case of the combination of the *SPINK 1* mutation and the *CTRC* CT genotype.

*The SPINK 1* mutation and *CTRC* polymorphism Hetero p.G60=; c.180C > T were equally frequent among the patients with disease relapses and without (2/111 vs. 4/188, *p* = 1 in the Fisher’s exact test).

## Discussion

In recent years, molecular studies have allowed us to identify mutations of key genes, including the *CTRC* gene, which might be crucial in the development of pancreatitis [16].

So far, only a few papers have been published that confirm the relationship of the *CTRC* gene polymorphism and an increased tendency to chronic pancreatitis, however, there is a lack of research in which patients with acute pancreatitis have taken part. Pathogenic *CTRC* variants were found both in the patients with CP and in the control group involving healthy individuals, thus, they should be considered risk factors, however, four *CTRC* variants have been recognized as pathogenic: p.A73T, p.V235I, p.R254W and p.K247_R254del [17].

Many authors observed a significant association of the synonymous variant c.180C > T (p.G60=) with CP [7, 16, 18, 19]. In the Indian studies, the variant c.180C > T (p.G60=) was found in 23–29% of the subject population. It increased the incidence risk by about 2.5 times in heterozygotes and tenfold in homozygotes [19]. In our paper, the analysis of the frequency distribution of the *CTRC* gene genotypes revealed a statistically significant correlation between the frequency of the CT (c.180C > T, p.G0=) genotype occurrence and developing acute pancreatitis (*p* < 0.05) with the adjusted odds ratio 1.6 (95% CI 1.1–2.2). What’s more, applying the four genetic models (Table 2), we have found that none of them have statistical significance. However, genotyping (TT vs. CC) and the dominant model (CT/TT vs. CC) are on the border of statistical significance.

Acute pancreatitis, especially recurring and chronic pancreatitis, should be treated as a complex disorder, in which various genetic and environmental factors can be of significance, which, when they co-occur, are responsible for increasing the risk of the disease development, a change in its character or a growing intensity of the developing process [20]. Tremblay et al. [21] conducted research in a group of 38 patients with acute pancreatitis and severe abdominal pain, as well as hypertriglyceridemia (Lipoprotein Lipase Deficiency- LPLD) and 100 healthy individuals (control). As a result of sequencing the *CTRC* and *SPINK1* genes, they found a positive correlation between the frequency of rehospitalization and a combination of the gene variants: rs545634 (IVS) (*CTRC*) – rs11319 (UTR-3’) (*SPINK1*) [*OR* = 41.4 (*CI*:2.0–848.0); *p* = 0.016]. LaRusch et al. [5] analyzed the influence of the variants *CTRC* p.G60 (c.180 T) on the risk of developing CP and RAP in a group of patients of European origin. Alcohol and tobacco smoking usually co-occurred, but the frequency of *CTRC* c.180 T in CP, but not in RAP, was higher among non-drinking smokers (10,8%), which suggests that smoking, not alcohol, may play a more significant role in this dependency. A frequent variant *CTRC* c.180 T may have an influence on the modifications of progression from RAP to CP, especially in patients with the *CFTR* or *SPINK1* mutations, who drink alcohol or smoke tobacco [5].

The studies on NAPS2 = CV showed that in the group of 521 patients with CP, there were 25,7% of patients who had never had AP. A higher percentage of patients with RAP never consumed alcohol or smoked cigarettes compared to the CP and control groups [5]. In our study, alcohol was the dominant cause of developing the disease in patients with polymorphism Homo c.180 T *CTRC* (60%). Alcohol was also a frequent cause of the AP relapses in patients with the c.180 T genotype (66,7%).

LaRusch et al. [5] found that *CTRC* c.180C > T alleles significantly increase the CP risk, but not RAP, which corresponds to the results of our study, in which no correlation was found between the disease relapses with *CTRC* c.180C > T and polymorphism Homo (c.180 T; p.G60=). In our study we did not confirm the relationship between *CTRC* c.180C > T and the *SPINK 1*, *CTRC* polymorphisms and the frequency of AP development, recurrence and an identified cause of the disease. LaRusch et al. [5] and Masson et al. [22] reported such a dependency in reference to the patients with CP. However, a new finding was the fact that the mutation *SPINK1* (hetero p.N34S), in combination with *CTRC* c.180C > T, can be responsible for a more severe course of acute pancreatitis. Due to the small number of patients (6 people), one cannot formulate a decisive conclusion and thus further research is necessary in order to confirm this dependency.

There is a growing number of studies which reveal that genetic factors together with environmental factors may increase the risk of developing pancreatitis.

## Conclusions

*CTRC* polymorphism Hetero p.G60=; c.180C > T increases the risk of developing acute pancreatitis, and in combination with the *SPINK1* mutation, can be responsible for a more severe course of the disease.

## Abbreviations

AP: Acute pancreatitis
ASA-PCR: Allele-specific PCR
BMI: Body mass index
*CASR*: The calcium-sensing receptor gene
*CFTR*: The cystic fibrosis transmembrane conductance regulator gene
*CLDN2*: The protein claudin-2
CP: Chronic pancreatitis
CPA: Carboxypeptydases A variants
CT: Images: computed tomography
*CTRC*: The chymotrypsinogen gene
ERCP: Endoscopic retrograde cholangiopancreatography
*GGT1*: Gammaglutamyltransferase1 gene
HRM: High resolution melting
ICP: Idiopathic chronic pancreatitis
NMR: Nuclear magnetic resonance
*PRSS1*: Cationic trypsinogen gene
*PRSS2*: Anionic trypsinogen
RAP: Recurrent acute pancreatitis
SADD: Short Alcohol Dependence Data
SAP: Severe acute pancreatitis
*SPINK1*: The pancreatic secretory trypsin inhibitor gene
USG: Ultrasonography.
